# Expression of a SOX1 overlapping transcript in neural differentiation and cancer models

**DOI:** 10.1007/s00018-017-2580-3

**Published:** 2017-07-03

**Authors:** Azaz Ahmad, Stephanie Strohbuecker, Cristina Tufarelli, Virginie Sottile

**Affiliations:** 1 Wolfson STEM Centre, Division of Cancer & Stem Cells, School of Medicine, University of Nottingham, Nottingham, UK; 2 Division of Graduate Entry Medicine & Health, School of Medicine, University of Nottingham, Derby, UK

**Keywords:** Stem cell, LncRNA, *SOX1*-*OT*, Expression pattern, Cancer model, *SOX1*

## Abstract

SOX1 is a member of the SOXB1 subgroup of transcription factors involved in early embryogenesis, CNS development and maintenance of neural stem cells. The structure and regulation of the human *SOX1* locus has been less studied than that of *SOX2*, another member of the SOXB1 subgroup for which an overlapping transcript has been reported. Here we report that the *SOX1* locus harbours a *SOX1* overlapping transcript (*SOX1*-*OT*), and describe expression, splicing variants and detection of *SOX1*-*OT* in different stem and cancer cells. RT-PCR and RACE experiments were performed to detect and characterise the structure of *SOX1*-*OT* in neuroprogenitor cultures and across different cancer cell lines. *SOX1*-*OT* was found to present a complex structure including several unannotated exons, different transcript variants and at least two potential transcription start sites. *SOX1*-*OT* was found to be highly expressed in differentiated neural stem cells across different time points of differentiation, and its expression correlated with *SOX1* gene expression. Concomitant expression of *SOX1* and *SOX1*-*OT* was further observed in several cancer cell models. While the function of this transcript is unknown, the regulatory role reported for other lncRNAs strongly suggests a possible role for *SOX1*-*OT* in regulating *SOX1* expression, as previously observed for *SOX2*. The elucidation of the genetic and regulatory context governing *SOX1* expression will contribute to clarifying its role in stem cell differentiation and tumorigenesis.

**Electronic supplementary material:**

The online version of this article (doi:10.1007/s00018-017-2580-3) contains supplementary material, which is available to authorized users.

## Introduction

**Fig. 1:**
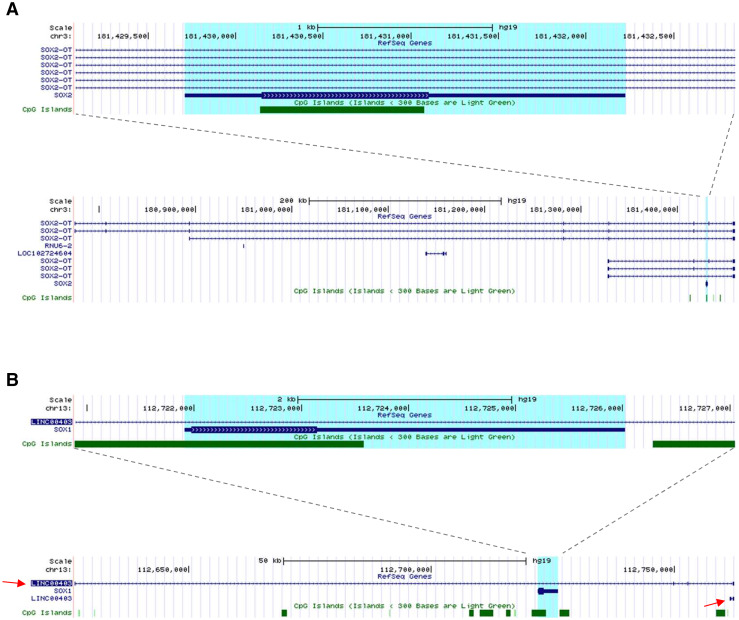
Structural similarity between SOX2 and SOX1 loci. **a** Snapshot images of the human *SOX2* locus on human chromosome 3 taken from the UCSC genome browser showing the *SOX2* gene itself (*top*) and the zoomed out region (*below*) to emphasise the length and alternative isoforms of the *SOX2*-*OT* non-coding gene within which *SOX2* lies. **b** Snapshots of the human *SOX1* locus on human chromosome 13 taken from the UCSC genome browser showing that similarly to SOX2, the *SOX1* gene is annotated within a larger non-coding gene (LINC00403, *top panel*), and that there are two isoforms for this gene currently annotated (*bottom panel*, *red arrows*). The regions highlighted in *blue* in **a** and **b** are the SOX2 and SOX1 genes, respectively

SOX1 and SOX2 are two closely related transcription factors belonging to the SOXB1 subgroup of the high mobility group box (HGM-box) family greatly involved in the regulation of pluripotent stem cells and neural stem cells [1]. In human, the *SOX2* gene maps to Chr3q26.3, within an intron of a long non-coding RNA (LncRNA) called *SOX2* overlapping transcript (*SOX2*-*OT*; Fig. 1a) [2]. LncRNAs, defined as non-coding RNAs (ncRNAs) that are more than 200 nucleotides long, have been suggested to play a role in several biological processes including nuclear organisation, epigenetic regulations and post-translational modifications [3, 4]. This structure is conserved between mouse and human, and in both species the *SOX2* overlapping transcripts are reported to have multiple transcription start sites (TSS) and to be transcribed into several alternative transcript variants [5]. Recently, concomitant gene expression of *SOX2* and *SOX2*-*OT* has been reported in breast, lung and oesophageal carcinoma [6–8]. Current studies suggest a positive role for *SOX2*-*OT* in regulating *SOX2*, and concordant gene expression has been reported in cellular differentiation, pluripotency and carcinogenesis [5–7, 9]. *SOX2*-*OT* is differentially spliced into multiple transcript variants in stem and cancer cells, and has been proposed to play a role in regulating expression of *SOX2* [9, 10].

*SOX1*, another SOXB1 member closely related to *SOX2*, is involved in early embryogenesis, CNS development and maintenance of neural stem cells [11]. SOX1 and SOX2 originated from a common ancestor by gene duplication during the course of evolution and exhibit similar sequences, expression patterns and overexpression phenotypes [12]. The structure and regulation of the human *SOX1* locus has been studied far less than that of *SOX2*, and there has been no report of any overlapping transcript for this gene. Here we address this question and describe the complex structure of the *SOX1* locus which was found to harbour an overlapping transcript, and describe expression, splicing variants and detection in different stem cell and cancer cell models.

## Materials and methods

Reagents were purchased from ThermoFisher (UK) unless otherwise stated.

### Cell sample preparation

Cells lines used in this study are described in Table 1. Ntera2, hMSCs, HeLa, SH-SY5Y and HOS cell lines were grown in Dulbeco’s Modified Eagle Medium (DMEM) supplemented with 10% foetal calf serum (FCS), 1% l-glutamine, 1% non-essential amino acids and 0.5% Penicillin/streptomycin, and incubated in a humidified incubator in an atmosphere of 5% CO_2_ at 37 °C. Cell culture conditions for MDA-MB-361, MDA-MB-231 and T47D are defined in [13], CaCo2, HCT116 and MCF7 in [14], Hs578T in [15]. For RNA extraction, cell monolayers were washed with PBS, detached with 0.05% trypsin/EDTA and pelleted for 5 min. Cell pellets were stored in TRI reagent (Sigma-Aldrich) at −80 °C.

**Table 1:** Cell lines used in the experimental study

No.	Cell line	Tissue	Cell type	References
1	Ntera2	Testis	Pluripotent embryonal carcinoma	[16]
2	hMSCs	Bone marrow	Mesenchymal progenitors	[17]
3	ReN cells	Brain	Neural progenitors	[18]
4	HeLa	Cervix	Adenocarcinoma	[19]
5	SH-SY5Y	Bone marrow	Neuroblastoma	[20]
6	HOS	Bone	Osteosarcoma	[21]
7	CaCo2	Colon	Colorectal Adenocarcinoma	[22]
8	HCT116	Colon	Colorectal Carcinoma	[23]
9	MCF7	Mammary gland	Adenocarcinoma	[24]
10	MDA-MB-361	Mammary gland	Adenocarcinoma	[25]
11	MDA-MB-231	Mammary gland	Adenocarcinoma	[25]
12	Hs578T	Mammary gland	Carcinoma	[26]
13	T47D	Mammary gland	Ductal carcinoma	[27]

For neural differentiation samples, human immortalised neuroprogenitor cells (ReNcell Merck Millipore, referred to as ‘ReN’) were cultured according to manufacturer’s instructions. Cells were seeded on laminin (Trevigen) in ReNcell NSC Maintenance Medium (Merck Millipore) supplemented with 20 ng/mL FGF2 and 20 ng/mL EGF. After 24 h incubation (day 0, D0), cells were treated with medium deprived of FGF and EGF to induce differentiation for up to day 6 (D6). At stated time points, RNA was harvested and processed as described below.

### Immunostaining

Cells were fixed with 4% paraformaldehyde (PFA), washed with PBS and incubated in PBS + 0.1% Triton X-100 for 10 min before blocking with a 10% serum dilution. After PBS wash, samples were incubated with a dilution of primary antibody against NESTIN (1:100, cat. # IC1259F, R&D Systems, Abingdon, UK) or MAP2 (1:100, MT-01, ExBio, Vestec, Czech Republic) overnight at 4 °C, before extensive washing, incubation in a FITC-labelled secondary antibody solution (1:500, FI-2000, Vector laboratories, Peterborough, UK) for 1 h at room temperature, further washing and finally mounting with DAPI-containing Vectashield (H-1200, Vector laboratories).

### RNA extraction

Total RNA was extracted using 0.5 mL of TRI-Reagent (Sigma-Aldrich) per 1–5 × 10^6^ cells according to the manufacturer’s protocol, followed by RNA purification from the aqueous phase using the RNA Clean & Concentrator-25 kit (Zymo Research). RNA concentration was determined spectrophotometrically and the samples were stored at −80 °C.

### cDNA synthesis and RT-PCR

RNA samples were subjected to DNAse-I treatment using the DNase-I, Amplification grade kit according to the manufacturer’s protocol, using 1 U/µL of DNase-I for each 1 µg of RNA at 25 °C for 20 min. After DNase-I treatment, 2 µg RNA was used to synthesise cDNA by reverse transcription using 200 units/µL of SuperScript III Reverse Transcriptase in 30 µL of total reaction volume, including 100 pmol/µL of random 15mer primers (MWG Biotech), 0.5 mM dNTP and 0.1 mM DTT. Tubes containing the reaction mix without reverse transcriptase (‘−RT’) were used as negative control. cDNA samples were cleaned up using MinElute PCR purification kit (Qiagen) and stored at −20 °C.

PCR amplification of cDNA was performed in a volume of 20 µL using Platinum Taq DNA polymerase. Thermal cycler conditions used after heating at 95 °C for 10 min involved 40 cycles of denaturation at 95 °C for 30 s, annealing at 55 °C for 60 s and extension at 72 °C for 60 s, followed by a final 7-min extension step at 72 °C. PCR reactions set using either water instead of cDNA (‘H_2_O’) or −RT samples as template were used as controls to rule out any contamination issues.

PCR products were analysed by electrophoresis on 2% agarose gels. Primers used for the *SOX1*-*OT* amplification are shown in Supplementary Table 1. All fragments detected by RT-PCR were sequenced (Source BioScience, Nottingham, UK) to confirm specificity and map their position.

### Quantitative polymerase chain reaction (qRT-PCR)

For gene quantification by real-time PCR, Taqman qPCR assays were performed in 20 µL reaction volumes containing 10 µL Taqman Gene Expression Master Mix (Applied Biosystems), 1 µL Taqman gene expression assay and 5 µL distilled water. Taqman assays used were Ref. Hs01057642_s1 for *SOX1* and three reference genes *YWHAZ* (Ref: Hs03044281_g), *GAPDH* (Ref: Hs02758991_g1) and *HPRT1* (Ref: Hs02800695_m1). qPCR was performed on an Applied Biosystem Fast 7500, with 50 cycles including a hold stage at 94 °C for 5 min followed by denaturation step at 94 C for 30 s and then annealing at specific primer temperatures for 45 s, followed by extension at 72 C for 1 min.

### Statistical analysis

For relative gene quantification of *SOX1* mRNA at different time points of neural differentiation, qPCR Ct values were normalised to the geometric mean of those of three reference genes (*GAPDH*, *HPRT1* and *YWHAZ*) according to MIQE guidelines [28]. Fold changes in gene expression were normalised to ReN cells at day 0 (2^−ΔΔCt^). One-way ANOVA with post hoc Tukey test was carried out for multiple comparison. Three technical replicates were used (*n* = 3), *p* value obtained <0.0001, 95% confidence interval, error bars represent ±RQ, Statistical software Graphpad prism 6 was used: ***p < 0.001; ****p < 0.0001.

### Bioinformatics analysis

The UCSC Blat [29] was used for the alignment of RT-PCR/5′RACE product sequences and the Blat results were then visualised using UCSC genome browser (https://genome.ucsc.edu) [30], Human Feb. 2009 (GRCh37/hg19) assembly [31]. FANTOM5 project tracks (http://fantom.gsc.riken.jp/5/) provided the TSS activities in individual biological states and the regions identified by CAGE (Cap Analysis Gene Expression) [32]. The ECR browser (http://ecrbrowser.dcode.org/) [33] was used to visualise and analyse Evolutionary Conserved Regions (ECRs) of *SOX1*-*OT* sequence across different species.

For RNA sequencing analysis, publicly available datasets were downloaded from the European nucleotide archive (ENA, http://www.ebi.ac.uk/ena). Paired-end RNA-seq [non-stranded, Poly(A)-enriched] was obtained in biological triplicates for a neural differentiation from H1 human neural progenitors on day 0, 1, 2, 4, 5, 11 and 18 (Array Express: E-GEOD-56785) [34]. After trimming the data using Trimmomatic [35] (first 10 bp, quality trimming), the reads were mapped to the human reference (Ensembl GRCh38) using HISAT2 [36]. For each sample, the transcriptome was assembled using StringTie [37] imposing a 2 read minimum for each splice site. The obtained transcriptomes were merged for the biological replicates and visualised using the IGV browser [38].

### 5′RACE

5′RACE experiments were carried out using the 5′RACE System for Rapid Amplification of cDNA Ends, version 2.0. All the steps were carried out according to manufacturer’s protocol using 2.5 µg DNAase I digested RNA from ReN cells differentiated at day 6, and *SOX1*-*OT*-specific primer pairs were GSP1, GSP2 and GSP3 with an annealing temperature of 60 °C (sequences available upon request). 5′RACE products were gel-purified with a gel extraction kit (Qiagen) and cloned using the TA cloning kit (Promega) according to the manufacturer’s instructions. Positive clones were analysed by Sanger sequencing (Source Biosciences); sequences obtained were aligned to the UCSC Genome Browser on Human Feb 2009 (GRCh37/hg19) Assembly.

## Results

### Comparative analysis of human and mouse *Sox1*-*OT* structure

Human *SOX2* and *SOX1* loci were analysed and revealed a high structural similarity (Fig. 1). Similar to *SOX2*, *SOX1* is also embedded within an intron of a LncRNA gene (referred hereafter as *SOX1*-*OT*), LINC00403, annotated in the NCBI RNA reference sequence collection (RefSeq) [30]. Two transcript variants were found annotated in RefSeq data, LINC00403 v1 and LINC00403 v2, but only transcript v1 showed overlap with the *SOX1* gene (Fig. 1b). LINC00403 is annotated as a 135.706 genomic region found on human chromosome 13: 112626624-112762329, giving rise to a 704 bp long RNA [30]. The LINC00403 structure has a validated status in RefSeq, and the reference sequences were derived from three different tissues, amygdala (GenBank: DA195709.1), foetal eye (GenBank: BQ184460.1.1) and Lung-carcinoid (GenBank: AI693652.1).

**Fig. 2:**
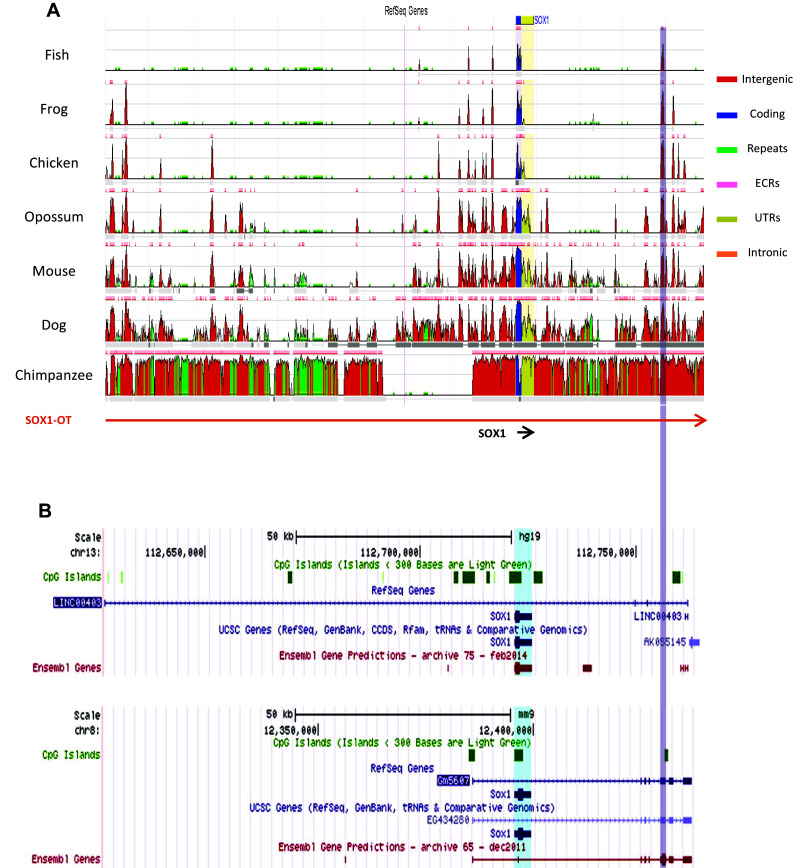
Cross-species comparative analysis of SOX1 overlapping transcript loci. **a** Evolutionary conserved regions revealed in the cross-species alignment of human assembly hg19 region chr13:112626600–112765500 generated by the ECR browser (http://ecrbrowser.dcode.org). *ECR* evolutionary conserved region, *UTR* untranslated region. **b** Snapshot images of the SOX1 overlapping transcript loci on human (hg19 chr13:112626600–112765500, *top panel*) and mouse (mm9, chr8: 12,300,135–12,439,035) taken from the UCSC genome browser to show the currently annotated structures of these transcripts in the two species. The conserved region highlighted in grey corresponds to an annotated exon in the mouse Sox1 overlapping transcript Gm5607 but not in human LINC00403. Note that the human gene AK055145 annotated 3′ to LINC0403 partly overlaps the 3′end of the mouse Gm5607 which extends further than the human transcript

Given the evolutionary conservation of the human *SOX2*-*OT* [5], multiple sequence alignment of the annotated human *SOX1*-*OT* genomic locus against different vertebrate species was carried out to evaluate the level of evolutionary conservation of the transcript (Fig. 2a) [33]. The comparative sequence alignment revealed some evolutionary conserved regions (ECR) across different vertebrate species, including an ECR towards the 3′end of *SOX1*-*OT* corresponding to an exon of the annotated mouse *Sox1* overlapping transcript GM5607 that was not found in the human annotation (Fig. 2b). Human–mouse alignment of this region demonstrated high level of sequence conservation (>99%; Supplementary Fig. 1A), which allowed the design of primers compatible with both human and mouse templates for experimental validation. RT-PCR performed using these primers confirmed that this region was expressed in mouse embryonic and neural stem cells (mESC and mNSC, respectively), and also revealed its expression in human cells with neural differentiation potential (NTera, ReN, SH-SY5Y) (Supplementary Fig. 1B, C). The detection of this yet unannotated exon, together with the presence of the several ECR highlighted by the cross-species comparison, suggested a possible conserved role for this transcript.

### Structural architecture of *SOX1*-*OT* in ReN cells

**Fig. 3:**
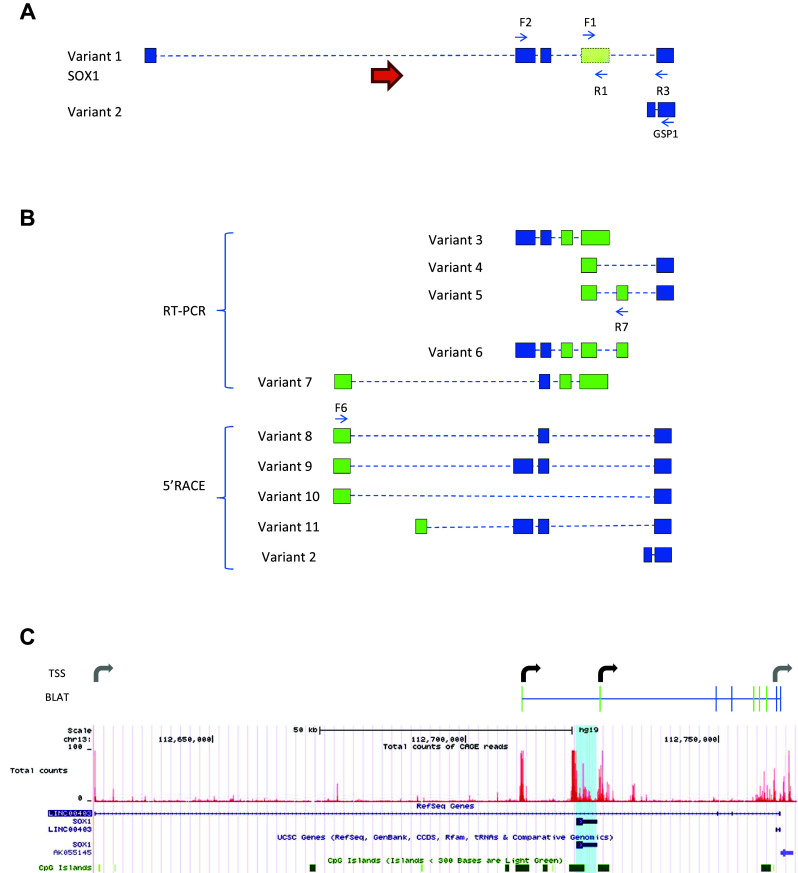
*SOX1*-*OT* structure in ReN human neuroprogenitor cells. **a** Schematic diagram showing *SOX1*-*OT* Variants 1 and 2 as annotated in the UCSC human genome browser, showing the evolutionary conserved region identified by cross-species comparison (*shaded green box*), the positions (*blue arrows*) of the RT-PCR (F1, R1, F2 and R3) and 5′RACE (GSP1) primers, and the location of *SOX1* (*orange arrow*). **b** Transcript variants identified by RT-PCR and 5′RACE. Variants 3–5 were identified by RT-PCR using primers within the conserved sequence (primers F1 and R1) in combination with primers in annotated exons; variant 6 was amplified using primer R7 in one of the newly identified exons from variant 6 and primer F2, while variant 7 was amplified using primer F6 in the first exon upstream of SOX1 identified by 5′RACE (variants 8, 9 and 10). 5′RACE products were amplified by anchoring the 5′RACE in the most 3′ exons of the annotated LINC0403 (primer GSP1 in **a**). **c** UCSC image of hg19 chr13:112626600–112765500 showing the *SOX1*-*OT* sequences detected in ReN cells sequence (BLAT, *top*) and the total count of CAGE reads (*red peaks*) from the FAMTON5 project track (*bottom*). *Bent arrows* indicate transcription start sites (TSS) either newly identified (*black*) or previously annotated (*grey*). For simplicity, diagrams in **a** and **b** are not to scale

Based on the initial strong signal for *SOX1*-*OT* in ReN cells, these cells were further used to characterise the structure of *SOX1*-*OT* using two parallel and complementary approaches: RT-PCR using primers in annotated exons of *SOX1*-*OT*, and 5′RACE to identify the transcription start site (TSS) of *SOX1*-*OT* (Fig. 3). RT-PCR revealed the presence of three new exons (in green in variants 3–6, Fig. 3b). 5′RACE primed in the last annotated exon uncovered two additional exons at the 5′ end of the transcript (in green in variants 8–11, Fig. 3b), the furthest 5′ of which was further validated by RT-PCR (variant 7, Fig. 3b).

The 5′RACE analysis revealed two main TSS for *SOX1*-*OT* located in close genomic proximity to the *SOX1* gene (Fig. 3c, bent arrows). To confirm the regulatory transcriptional potential of these two TSS, an online bioinformatics analysis was performed by aligning the *SOX1*-*OT* sequence to the FANTOM5 project tracks through the UCSC genome browser (Fig. 3c) [39]. The FANTOM5 project provides genome-wide mammalian gene expression data by mapping TSS, promoter regions and enhancers in human and mouse primary cells, cell lines and tissues [32]. The alignment highlighted two potential transcriptional start sites with high peaks of Cap analysis for gene expression (CAGE) reads that matched with the TSS experimentally identified by 5′RACE, providing further support for the identity of the *SOX1*-*OT* TSS found in ReN cells (Fig. 3c, red tracks).

### *SOX1*-*OT* and *SOX1* are co-expressed during neural differentiation

**Fig. 4:**
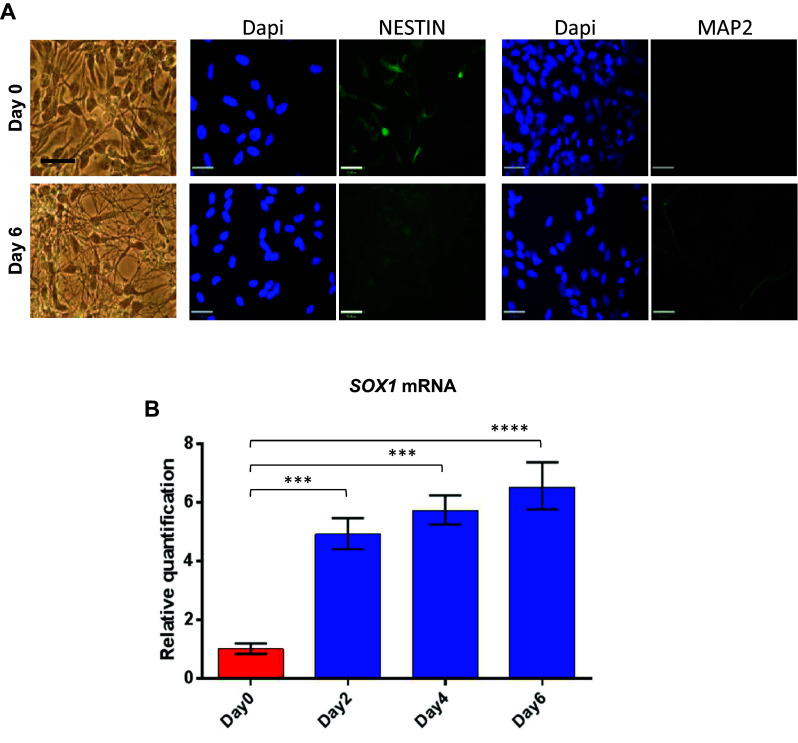
Differentiation of the ReN human neuroprogenitor cell line. **a** Imaging of day 0 (control) and day 6 (differentiated) ReN cultures in brightfield (*left panels*) and following immunostaining for NESTIN and MAP2 (*green*) with corresponding dapi counterstain (*blue*). *Bar* 50 µm. **b** Relative quantification (RQ) of *SOX1* mRNA expression analysed by quantitative RT-PCR across the different time points of ReN cells differentiation (day 0, 2, 4 and 6). *Error bars* represent ±RQ, *n* = 3, ***p < 0.001; ****p < 0.0001

To determine whether the different *SOX1*-*OT* variants were expressed at different levels during neuronal differentiation, ReN neuroprogenitor cells were differentiated over a 6-day time course, and tested as undifferentiated (D0) or after 2, 4 and 6 days of neural differentiation. ReN cell differentiation was confirmed by immunofluorescence at D0 and D6 showing loss of the undifferentiated marker Nestin and increase in MAP2 expression (a neuronal marker) (Fig. 4a). Relative quantification of *SOX1* expression in ReN cells over the 6-day differentiation showed that *SOX1* mRNA significantly increased at D2, D4 and D6 of differentiation compared to D0 (Fig. 4b).

**Fig. 5:**
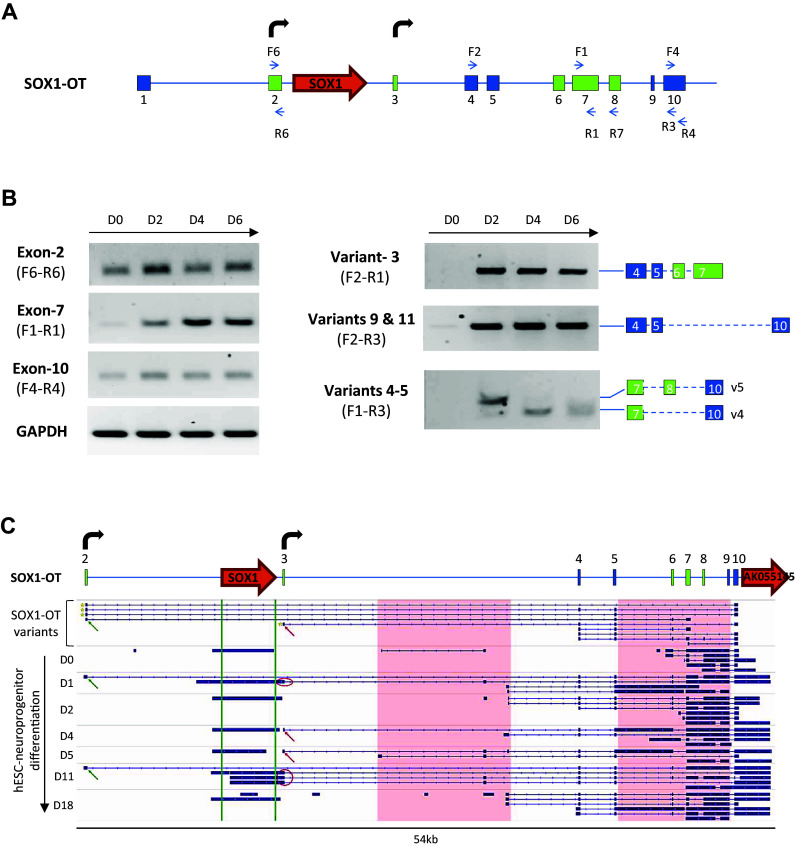
Expression profile of *SOX1*-*OT* during human neuroprogenitor differentiation. **a** Composite structure of the *SOX1*-*OT* genomic locus including the exons (*green boxes*) and additional TSS (*bent arrows*) newly identified in ReN cells; primers used in the expression profiling are shown as *blue arrows*. **b** RT-PCR detection of *SOX1*-*OT* exons 2, 7, 10 and transcript variants 3, 4, 5, 9, 11 in undifferentiated ReN cells (D0) and at day 2 (D2), 4 (D4) and 6 (D6) of neural differentiation. GAPDH was used as positive control for RT reaction. **c** Schematic diagram of the *SOX1*-*OT* locus in ReN cells (*top*) with indicated exons (*numbered boxes*) and TSS (*bent arrows*), and IGV visualisation of the transcriptome assembly (*bottom*) for neural differentiation time course of human embryonic stem cell-derived neuroprogenitor cells compared to the *SOX1*-*OT* isoforms identified in this study (*top row*). *Green* and *red arrows* show TSS detected using 5′RACE; the Sox1 region is shown between *green vertical lines*; *pink boxes* show regions which differ between the *SOX1*-*OT* isoforms detected in H1 hESC vs ReN cells; *red circles* highlight SOX1 isoforms that merge with *SOX1*-*OT* isoforms. Note that some of the transcripts in H1 hESC suggest that AK055145 is part of the *SOX1*-*OT* transcript

*SOX1*-*OT* expression was analysed in these cells using different primer pairs to test selected individual exons and different transcript variants (Fig. 5a). Expression of exons 2, 7 and 10 was detected at all time points of differentiation, with a significant increase in expression between D0 and D2 for all exons tested (Fig. 5b, left panel). Similarly, transcript variants 3, 4, 5, 9 and 11 were expressed at very low levels (variants 9 and 11) or not detected (variants 3, 4 and 5) in undifferentiated ReN cells (D0), but became detectable from D2. Of particular note, there seemed to be a switch in expression between variants 5 and 4, whereby variant 5 was expressed at D2 but not at D4 and D6, when variant 4 became more prominent (Fig. 5b). These results indicate that during neural differentiation of ReN cells, *SOX1*-*OT* expression pattern is similar to that of *SOX1*, with a significant increase in expression observed at day 2 and 4 for both genes. Transcriptome analysis of publicly available data for human embryonic stem cells (hESC H1)-derived neural progenitors over a 18-day neural differentiation time course supported the findings in ReN cells [34]. The two TSS identified in ReN cells were also found in differentiating hESC-derived neuroprogenitors, as well as many of the variants identified experimentally (Fig. 5c). Interestingly, this analysis revealed the existence of additional variants in differentiating hESC-derived neuroprogenitors, and also suggested that the gene AK055145 annotated just 3′ to the last exon of LINC00403 may be part of some *SOX1*-*OT* transcript variants in this cell type (Fig. 5c). This observation appeared to better mirror the data obtained for the annotated mouse *Sox1*-*ot* transcript Gm5607 (see human and mouse locus comparison in Fig. 2b). The analysis was extended to human neural tissue, through transcriptome analysis of RNA-seq data available for developing human cortex [40]. This analysis identified a greater variety of *SOX1*-*OT* transcript variants over increasing gestational time points in developing cortex tissue than in the cell samples used in this study (Supplementary Fig. 2). Nevertheless, many were found to initiate at a transcription start site (TSS) very close to, if not identical to, the TSS detected by 5′RACE. Evidence from this transcriptome analysis also suggested that *SOX1*-*OT* may extend further than its currently annotated 3′ end.

### Protein-coding gene AK55145 is part of *SOX1*-*OT*

**Fig. 6:**
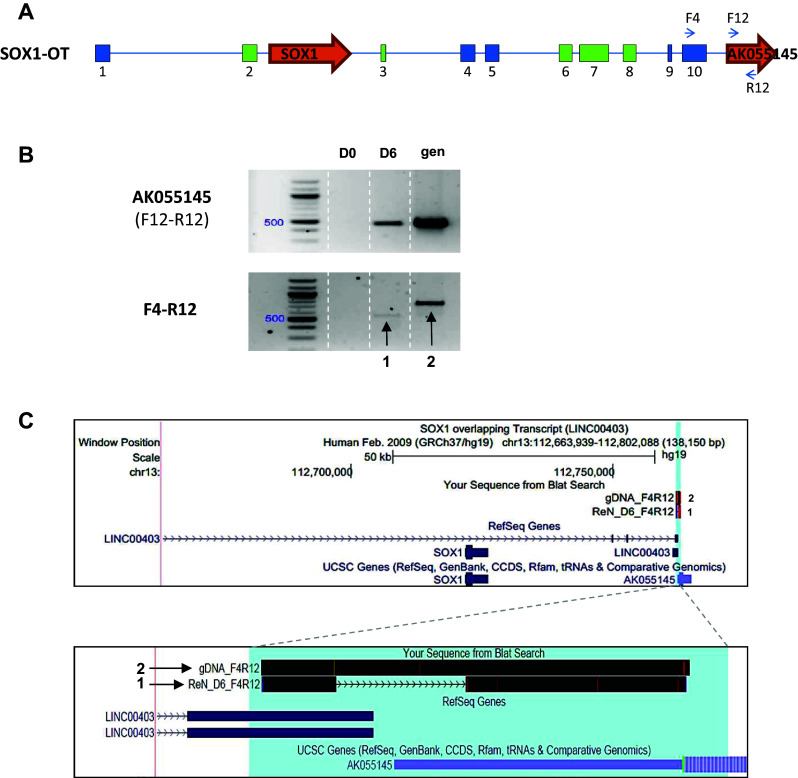
LINC00403 and AK055145 are part of *SOX1*-*OT*. **a** Schematic diagram showing the positions of primers used in exon 10 of SOX1OT and within the annotated gene AK055145. **b** RT-PCR product obtained using cDNA from ReN cells at day 0 (D0) and 6 (D6) of differentiation; genomic DNA (gen) was used as a positive control for PCR. PCR F12-R12 was co-linear to the genomic DNA and gave rise to a 462-bp product only detectable in D6 cells; F4–R12 amplicons spanned the region from exon 10 of LINC00403 and AK055145 with an expected genomic size of 762 bp (2); this primer pair detected a spliced product of 550 bp in D6 ReN cells (1) but not in D0. **c** Sequences obtained for genomic and D6 ReN cDNA PCR were aligned to the human genome assembly (GRCh37/hg19) using blat and visualised using the UCSC genome browser (*top box*); an enlargement of the blat alignment showed the D6 ReN product (1) spanned from LINC00403 through to AK055145

To further investigate the 3′ extent of the human *SOX1*-*OT* transcript, primers within AK055145 (F12 and R12, Fig. 6a) were used alone or in combination with primer F4 in the last annotated exon of LINC00403 to test expression in D0 and D6 ReN cells (Fig. 6a).

RT-PCR detection of AK55145 gene expression (primer pair F12–R12) showed that AK55145 was only detected in differentiated ReN cells (Fig. 6b, top panel). Using primer pair F4–R12, a product was amplified in D6 ReN cells suggesting that the last exon from *SOX1*-*OT* and the AK55145 gene may be part of the same transcript. To confirm our findings, the PCR fragments amplified from D6 cDNA and from gDNA were sequenced and aligned to the genome using BLAT [29], confirming that the *SOX1*-*OT* transcript extended to include the AK55145 gene. Therefore, these results indicated that the locus of *SOX1*-*OT* extends further downstream than the currently annotated *SOX1*-OT transcript as shown in the UCSC genome browser-generated images (Fig. 6c).

### *SOX1*-*OT* expression correlates with *SOX1* gene expression in different cancer cell lines

**Fig. 7:**
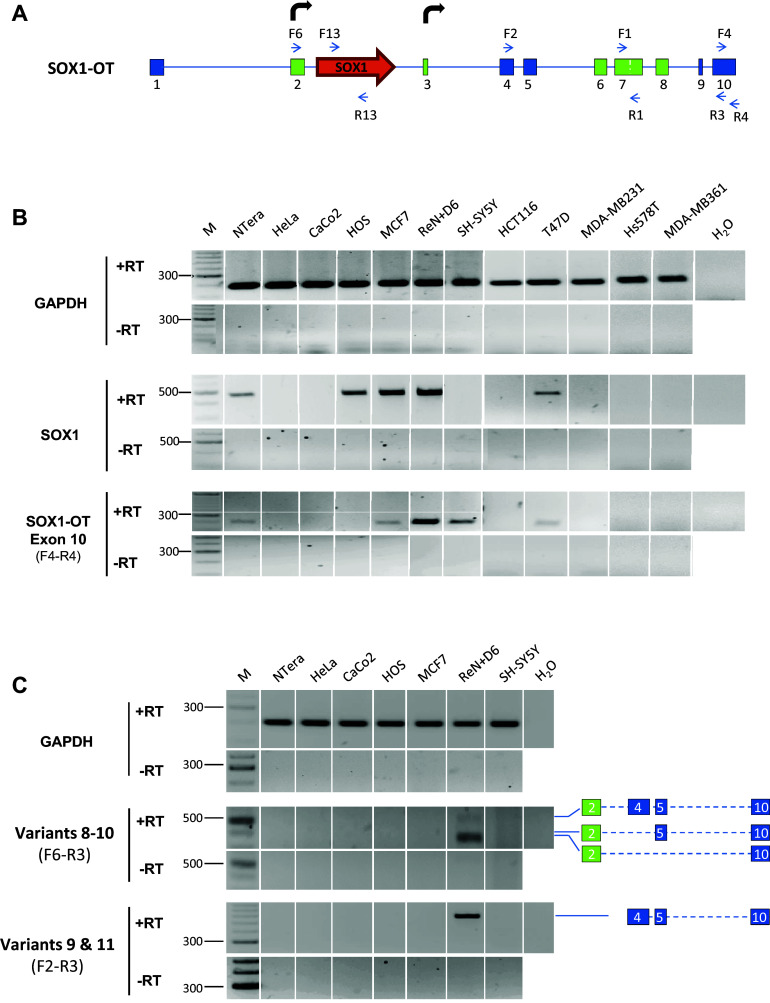
*SOX1* and *SOX1*-*OT* expression in a panel of human cancer cell lines. **a** Schematic diagram of the *SOX1*-*OT* locus showing the positions of the primer pairs used to detect *SOX1* and different regions of *SOX1*-*OT* in cancer cell lines. **b** RT-PCR detection of *SOX1* and *SOX1*-*OT* (exon 10) in the stated human cancer cell lines and in day 6 differentiated ReN cells as a positive control. **c** RT-PCR detection of *SOX1*-*OT* variants 8–11 and exon 7 detected with the indicated primer combinations in the stated human cell lines. PCRs performed on total RNA after reverse transcription with (+RT) or without (−RT) reverse transcriptase; *M* DNA size standard; *H*_*2*_ *O* no template PCR negative control

Recently, several reports have suggested *SOX1* involvement in cancer development [41–43], and the present study has investigated whether *SOX1* gene expression may correlate with expression of *SOX1*-*OT* in cancer. To achieve this, *SOX1* and *SOX1*-*OT* expression was analysed in a variety of cancer cell lines by RT-PCR using different combinations of primer pairs across the locus (Fig. 7a). Primer pair F4-R4 was used to detect the last annotated exon of LINC00403 that is shared by several *SOX1*-OT variants (see Fig. 3b). Expression of the *SOX1* amplicon (primers F13–R13) was co-detected with that of the *SOX1*-*OT* F4–R4 region in most of the cell lines analysed (Fig. 7B). *SOX1* and *SOX1*-*OT* were co-detected in teratocarcinoma (NTera) and some breast cancer (MCF7, T47D) cell lines, but not in colon (HCT116, CaCo-2), some breast (MDA-MB-231/361, Hs578T) and cervical (HeLa) cancer cells (Fig. 7b). The exception to this pattern was the osteoblast HOS cell line, which expressed the *SOX1* gene but not *SOX1*-*OT*, and the neuroblastoma SH-SY5Y cell line which presented the opposite pattern. Using primer pair F6–R3, we detected *SOX1*-*OT* variants 8–10 that span the *SOX1* gene, but no *SOX1*-*OT* variant spanning the SOX1 gene was detected in the cancer cell lines tested (Fig. 7c). Transcriptome tracks for HeLa and MCF7 cells available through the ENCODE project annotations were analysed and indicated patterns consistent with our RT-PCR results, showing HeLa cells negative throughout this region, while some transcription could be seen in MCF7 cells across the locus (Supplementary Fig. 3). These findings suggested that *SOX1*-*OT* variants spanning the *SOX1* gene are expressed in MCF7 cells, but these appeared to have a different structure to those found in ReN cells.

## Discussion

### Characterisation of the structure of *SOX1*-*OT*

Genome-wide studies have reported large numbers of non-coding RNAs whose function and significance are not clear. To understand the complex transcriptome architecture, expression and regulation of genetic information, it has become necessary to distinguish between mRNA and ncRNA transcripts [44]. Here we show that the *SOX1*-*OT* transcript, annotated as a long intergenic non-coding mRNA-like transcript with no inferred coding potential, could be detected in human cells. *SOX1*-*OT* has a complex structure including several unannotated exons, different transcript variants, and at least two potential TSS. Our data identified a total of 10 exons for human *SOX1*-*OT*, 5 of which (exon2, 3, 6–8) are novel and previously unknown. In addition to the two annotated transcript variants (V1–V2), we report 9 new *SOX1*-*OT* transcript variants (V3–V11) not previously reported in the literature. Therefore, *SOX1*-*OT* presents complex transcriptional features, whose potential functions and biological significance remain to be explored. The TSS identified for human *SOX1*-*OT* is located in close genomic proximity to and upstream of the *SOX1* gene (Fig. 1a), suggesting a possible role in regulating *SOX1* gene expression. The likelihood of *SOX1*-*OT* acting as a regulator of *SOX1* is supported by similarities with the *SOX2* locus. The multi-exon, non-coding *SOX2*-*OT* transcript overlapping *SOX2* has recently been shown to give rise to multiple splice variants from different TSS, and is attributed a positive regulatory role in *SOX2* transcription [9].

Our results show that the first exon of the RefSeq-annotated transcript LINC00403 is either absent or expressed at levels below the present detection limits in ReN cells. However, it is important to note that the current annotated structure of *SOX1*-*OT* has been obtained by combining information collected from three different tissues types (amygdala, eye, carcinoid); this might explain the differences with the present study, which focused on characterising the transcript in a well-defined neural cell type. Interestingly, the newly experimentally characterised structure of human *SOX1*-*OT* resembles that of the annotated mouse *Sox1*-*ot*. Both have TSS upstream of and near to the *SOX1* coding gene; moreover, although the 3′ end of the mouse overlapping transcript extends further downstream compared to the current annotation of the human *SOX1*-*OT*, our findings extend human *SOX1*-*OT* to include the downstream AK55145 gene, in line with the mouse transcript 3′ end. Our results suggest this 3′ end might be used in differentiated ReN cells and not in undifferentiated cells; further work will be required to determine whether Transcription Termination End (TTE) usage is regulated in a cell type/tissue/differentiation stage specific manner.

### Potential role of *SOX1*-*OT* in neural differentiation as a regulator of *SOX1*

*SOX1*-*OT* was found to be highly expressed in differentiated neural stem cells, and its expression appeared to correlate with *SOX1* gene expression. Different *SOX1*-*OT* transcript variants were differentially detected during the course of neural differentiation. Our observed correlation between *SOX1* and *SOX1*-*OT* expression during neural differentiation is similar to that reported for *Sox2* and *Sox2*-*ot* during mouse neurosphere differentiation in vitro; however, in this case both *Sox2* and *Sox2*-*ot* were upregulated after day 2 and then slightly downregulated at day 7 of neural differentiation [5], while here *SOX1* and *SOX1*-*OT* were upregulated at day 2 and expression remained upregulated towards day 6 of neural differentiation in vitro. It is therefore possible that co-expression of *SOX1*-*OT* and *SOX1* during neural differentiation might indicate a co-regulatory role in pathways regulating neural differentiation. Furthermore, we observed a switch between transcript variants 4 and 5 from day 2 to 4, further supporting a possible regulatory role during neural differentiation. Further experiments testing the effect of forced expression or downregulation of the new transcript will be required to determine if *SOX1*-*OT* plays a functional role in neural differentiation and a possible link to *SOX1* expression.

### *SOX1*-*OT* and *SOX1* are concomitantly expressed in different cancerous cell lines

*SOX1* expression has been already reported in several cancer types [45–48]. We detected co-expression of *SOX1*-*OT* and *SOX1* RNAs in NTera, T47D and MCF7 cancer cell lines. Concomitant expression of *SOX2* and its LncRNA *SOX2*-*OT* has been described in different cancer types, and it was shown that *SOX2* gene expression is regulated by *SOX2*-*OT* in this context. For example, *SOX2*-*OT* is upregulated together with *SOX2* and *OCT4* in oesophageal squamous cell carcinoma [6]. Moreover, co-expression of *SOX2*-*OT* and *SOX2* has been previously reported in the NTera cell line, and *SOX2*-*OT* has been functionally associated with the *SOX2* gene in pluripotency and tumorigenesis [9]. Also, concordant expression of *SOX2* and *SOX2*-*OT* has been reported in breast cancer and both are upregulated in cell suspension culture conditions that favour stem cell expansion [7].

Our finding of *SOX1*-*OT* expression in the NTera cell line, which possesses stem cell-like properties, indicates a potential role of *SOX1*-*OT* in pluripotency and cancer development. Similar to *SOX2* and *SOX2*-*OT*, expression of *SOX1*-*OT* and *SOX1* in breast cancer cell lines (MCF7 and T47D) also suggests a possible co-regulatory role in breast cancer.

Therefore, *SOX1*-*OT* might have a potential role in cancer by promoting *SOX1* expression; its expression in different cancer types in which *SOX1* has already been reported will need further investigation. Based on our data and in silico analysis, it is possible that cells from different tissues and/or different cell types from the same tissue may express different repertoires of transcript variants, so further larger scale expression analyses will be required to identify all the isoforms, their structures and polyA sites. Indeed, analysis of Poly-A seq data from both mouse and human brain samples confirmed the presence of a variability of poly-A signals (Suppl. Figure 4). In the context of cancer, the structure of alternative SOX1-OT variants expressed in cancer types that express SOX1 will also require consideration, in order to identify the repertoire of transcript variants expressed through large-scale gene expression and RACE analyses. Interestingly, the osteosarcoma cell line HOS has been shown to express *SOX1* but not *SOX1*-*OT*, while in contrast the neuroblastoma cell line SH-SY5Y showed signal for *SOX1*-*OT* but not *SOX1*. This observation indicates that *SOX1* and *SOX1*-*OT* expression might be independent of each other in these cancer types, or there might be another regulatory mechanism for these two transcripts, which requires further exploration.

Our results also indicate that the *SOX1*-*OT* locus extends further downstream than the currently annotated *SOX1*-*OT* transcript, suggesting that the gene AK055145 annotated just 3′ to the last exon of LINC00403 may be part of some *SOX1*-*OT* transcript variants. Therefore, further experiments such as 3′RACE will be necessary to confirm this initial observation.

## Conclusion

In conclusion, we report the expression of an overlapping transcript at the *SOX1* locus, and have demonstrated that *SOX1*-*OT* has a complex structure with two potential TSS and multiple transcript variants. These transcript variants are highly expressed in differentiating neuroprogenitors, where their expression coincides with that of *SOX1*. Furthermore, we have shown co-expression of *SOX1*-*OT* and *SOX1* RNA in neural and cancer cell lines, suggesting a possible role for *SOX1*-*OT* in stem cell differentiation and cancer. Further work is now needed to determine the function of *SOX1*-*OT* and its potential regulatory link to *SOX1* expression.

### Electronic supplementary material

Below is the link to the electronic supplementary material.

Supplementary material 1 (DOCX 34 kb)

Supplementary material 2 (PDF 75 kb)

Supplementary material 3 (PDF 163 kb)

Supplementary material 4 (PDF 71 kb)

Supplementary material 5 (PDF 96 kb)

## References

[1] Zhang S, Cui W (2014). Sox2, a key factor in the regulation of pluripotency and neural differentiation. World J Stem Cells.

[2] Fantes J, Ragge NK, Lynch SA, McGill NI, Collin JR, Howard-Peebles PN (2003). Mutations in SOX2 cause anophthalmia. Nat Genet.

[3] Quinodoz S, Guttman M (2014). Long noncoding RNAs: an emerging link between gene regulation and nuclear organization. Trends Cell Biol.

[4] Cao J (2014). The functional role of long non-coding RNAs and epigenetics. Biol Proced Online.

[5] Amaral PP, Neyt C, Wilkins SJ, Askarian-Amiri ME, Sunkin SM, Perkins AC, Mattick JS (2009). Complex architecture and regulated expression of the Sox2ot locus during vertebrate development. RNA.

[6] Shahryari A, Rafiee MR, Fouani Y, Oliae NA, Samaei NM, Shafiee M (2014). Two novel splice variants of SOX2OT, SOX2OT-S1, and SOX2OT-S2 are coupregulated with SOX2 and OCT4 in esophageal squamous cell carcinoma. Stem Cells.

[7] Askarian-Amiri ME, Seyfoddin V, Smart CE, Wang J, Kim JE, Hansji H (2014). Emerging role of long non-coding RNA SOX2OT in SOX2 regulation in breast cancer. PLoS One.

[8] Güre AO, Stockert E, Scanlan MJ, Keresztes RS, Jäger D, Altorki NK (2000). Serological identification of embryonic neural proteins as highly immunogenic tumor antigens in small cell lung cancer. Proc Natl Acad Sci USA.

[9] Shahryari A, Jazi MS, Samaei NM, Mowla SJ (2015). Long non-coding RNA SOX2OT: expression signature, splicing patterns, and emerging roles in pluripotency and tumorigenesis. Front Genet.

[10] Saghaeian Jazi M, Samaei NM, Ghanei M, Shadmehr MB, Mowla SJ (2016). Identification of new SOX2OT transcript variants highly expressed in human cancer cell lines and down regulated in stem cell differentiation. Mol Biol Rep.

[11] Kan L, Israsena N, Zhang Z, Hu M, Zhao LR, Jalali A (2004). Sox1 acts through multiple independent pathways to promote neurogenesis. Dev Biol.

[12] Archer TC, Jin J, Casey ES (2011). Interaction of Sox1, Sox2, Sox3 and Oct4 during primary neurogenesis. Dev Biol.

[13] Huguet EL, McMahon JA, McMahon AP, Bicknell R, Harris AL (1994). Differential expression of human Wnt genes 2, 3, 4, and 7B in human breast cell lines and normal and disease states of human breast tissue. Cancer Res.

[14] Cruickshanks HA, Vafadar-Isfahani N, Dunican DS, Lee A, Sproul D, Lund JN (2013). Expression of a large LINE-1-driven antisense RNA is linked to epigenetic silencing of the metastasis suppressor gene TFPI-2 in cancer. Nucleic Acids Res.

[15] Colston KW, Perks CM, Xie SP, Holly JM (1998). Growth inhibition of both MCF-7 and Hs578T human breast cancer cell lines by vitamin D analogues is associated with increased expression of insulin-like growth factor binding protein-3. J Mol Endocrinol.

[16] Andrews PW, Damjanov I, Simon D, Banting GS, Carlin C, Dracopoli NC, Føgh J (1984). Pluripotent embryonal carcinoma clones derived from the human teratocarcinoma cell line Tera-2. Differentiation in vivo and in vitro. Lab Invest.

[17] France LA, Scotchford CA, Grant DM, Rashidi H, Popov AA, Sottile V (2014). Transient serum exposure regimes to support dual differentiation of human mesenchymal stem cells. Tissue Eng Regen Med.

[18] Hoffrogge R, Mikkat S, Scharf C, Beyer S, Christoph H, Pahnke J (2006). 2-DE proteome analysis of a proliferating and differentiating human neuronal stem cell line (ReNcell VM). Proteomics.

[19] Scherer WF, Syverton JT, Gey GO (1953). Studies on the propagation in vitro of poliomyelitis viruses. IV. Viral multiplication in a stable strain of human malignant epithelial cells (strain HeLa) derived from an epidermoid carcinoma of the cervix. J Exp Med.

[20] Biedler JL, Roffler-Tarlov S, Schachner M, Freedman LS (1978). Multiple neurotransmitter synthesis by human neuroblastoma cell lines and clones. Cancer Res.

[21] Rhim JS, Cho HY, Vernon ML, Arnstein P, Huebner RJ, Gilden RV (1975). Characterization of non-producer human cells induced by Kirsten sarcoma virus. Int J Cancer.

[22] Sambuy Y, De Angelis I, Ranaldi G, Scarino ML, Stammati A, Zucco F (2005). The Caco-2 cell line as a model of the intestinal barrier: influence of cell and culture-related factors on Caco-2 cell functional characteristics. Cell Biol Toxicol.

[23] Brattain MG, Fine WD, Khaled FM, Thompson J, Brattain DE (1981). Heterogeneity of malignant cells from a human colonic carcinoma. Cancer Res.

[24] Soule HD, Vazguez J, Long A, Albert S, Brennan M (1973). A human cell line from a pleural effusion derived from a breast carcinoma. J Natl Cancer Inst.

[25] Cailleau R, Olive M, Cruciger QV (1978). Long-term human breast carcinoma cell lines of metastatic origin: preliminary characterization. In Vitro.

[26] Hackett AJ, Smith HS, Springer EL, Owens RB, Nelson-Rees WA, Riggs JL, Gardner MB (1977). Two syngeneic cell lines from human breast tissue: the aneuploid mammary epithelial (Hs578T) and the diploid myoepithelial (Hs578Bst) cell lines. J Natl Cancer Inst.

[27] Keydar I, Chen L, Karby S, Weiss FR, Delarea J, Radu M (1979). Establishment and characterization of a cell line of human breast carcinoma origin. Eur J Cancer.

[28] Bustin SA, Benes V, Garson JA, Hellemans J, Huggett J, Kubista M, Mueller R, Nolan T, Pfaffl MW, Shipley GL (2009). The MIQE guidelines: minimum information for publication of quantitative real-time PCR experiments. Clin Chem.

[29] Kent WJ (2002). BLAT—the BLAST-like alignment tool. Genome Res.

[30] Kent WJ, Sugnet CW, Furey TS, Roskin KM, Pringle TH, Zahler AM, Haussler D (2002). The human genome browser at UCSC. Genome Res.

[31] Speir ML, Zweig AS, Rosenbloom KR, Raney BJ, Paten B, Nejad P (2016). The UCSC Genome Browser database: 2016 update. Nucleic Acids Res.

[32] FANTOM Consortium and the RIKEN PMI and CLST (DGT), Forrest AR, Kawaji H, Rehli M, Baillie JK, de Hoon MJ, et al. (2014) A promoter-level mammalian expression atlas. Nature 507:462-70 10.1038/nature13182 PMC4529748 24670764

[33] Ovcharenko I, Nobrega MA, Loots GG, Stubbs L (2004). ECR Browser: a tool for visualizing and accessing data from comparisons of multiple vertebrate genomes. Nucleic Acids Res.

[34] Sauvageau M, Goff LA, Lodato S, Bonev B, Groff AF, Gerhardinger C (2013). Multiple knockout mouse models reveal lincRNAs are required for life and brain development. Elife.

[35] Bolger AM, Lohse M, Usadel B (2014). Trimmomatic: a flexible trimmer for Illumina sequence data. Bioinformatics.

[36] Kim D, Langmead B, Salzberg SL (2015). HISAT: a fast spliced aligner with low memory requirements. Nat Methods.

[37] Pertea M, Pertea GM, Antonescu CM, Chang TC, Mendell JT, Salzberg SL (2015). StringTie enables improved reconstruction of a transcriptome from RNA-seq reads. Nat Biotechnol.

[38] Thorvaldsdottir H, Robinson JT, Mesirov JP (2013). Integrative Genomics Viewer (IGV): high-performance genomics data visualization and exploration. Brief Bioinform.

[39] Rosenbloom KR, Sloan CA, Malladi VS, Dreszer TR, Learned K, Kirkup VM (2013). ENCODE data in the UCSC Genome Browser: year 5 update. Nucleic Acids Res.

[40] Liu SJ, Nowakowski TJ, Pollen AA, Lui JH, Horlbeck MA, Attenello FJ (2016). Single-cell analysis of long non-coding RNAs in the developing human neocortex. Genome Biol.

[41] Guan Z, Zhang J, Wang J, Wang H, Zheng F, Peng J (2014). SOX1 down-regulates beta-catenin and reverses malignant phenotype in nasopharyngeal carcinoma. Mol Cancer.

[42] Song L, Liu D, He J, Wang X, Dai Z, Zhao Y (2016). SOX1 inhibits breast cancer cell growth and invasion through suppressing the Wnt/beta-catenin signaling pathway. APMIS.

[43] Mathews LA, Hurt EM, Zhang X, Farrar WL (2010). Epigenetic regulation of CpG promoter methylation in invasive prostate cancer cells. Mol Cancer.

[44] Dinger ME, Pang KC, Mercer TR, Mattick JS (2008). Differentiating protein-coding and noncoding RNA: challenges and ambiguities. PLoS Comput Biol.

[45] Tsao CM, Yan MD, Shih YL, Yu PN, Kuo CC, Lin WC (2012). SOX1 functions as a tumor suppressor by antagonizing the WNT/beta-catenin signaling pathway in hepatocellular carcinoma. Hepatology.

[46] Apostolidou S, Hadwin R, Burnell M, Jones A, Baff D, Pyndiah N (2009). DNA methylation analysis in liquid-based cytology for cervical cancer screening. Int J Cancer.

[47] Su HY, Lai HC, Lin YW, Chou YC, Liu CY, Yu MH (2009). An epigenetic marker panel for screening and prognostic prediction of ovarian cancer. Int J Cancer.

[48] Zhao Y, Zhou H, Ma K, Sun J, Feng X, Geng J (2013). Abnormal methylation of seven genes and their associations with clinical characteristics in early stage non-small cell lung cancer. Oncol Lett.

