# Supplementary material for: Expression of a SOX1 overlapping transcript in neural differentiation and cancer models
Source: Cell Mol Life Sci. 2017 Jul 3;74(22):4245–58. doi: 10.1007/s00018-017-2580-3 (PMC5641280; doi:10.1007/s00018-017-2580-3)
Supplement: Supplementary file 1 — Supplementary material 1 (DOCX 34 kb) [file 18_2017_2580_MOESM1_ESM.docx]

**ADDITIONAL INFORMATION**

**SUPPLEMENTARY FIGURE LEGENDS:**

**Supplementary Fig.1: Alignment highlights conserved sequence annotated as exon on mouse *Sox1-ot* also detected in human cells.** (**A**) Blast alignment of a 218bp fragment of the conserved region (highlighted in grey in Fig.2) for mouse (mm9 chr8:12,430,257-12,430,474) and human (hg19 chr13: 112,758,395-112,758,612). F1 (yellow) and R1 (green) indicate the location of the primers designed to amplify this fragment in human and mouse. (**B**) Resulting RT-PCR on mouse ES cells (mESC) either undifferentiated (day 0) or after 4 days neural differentiation (day 4), and in mouse neural stem cells (mNSC), showing expression of this exon (top panel). (**C**) Resuting RT-PCR on a panel of human cancer cell lines and differentiated neuroprogenitors (ReN+D6) showing that this exon is expressed preferentially in human cells with neural potential (NTera, ReN+D6, SH-SY5Y). PCRs performed on total RNA after reverse transcription with (+) or without (-) reverse transcriptase; M=DNA size standard; H_2_O=no template PCR negative control; gDNA=genomic DNA PCR positive control.

**Supplementary Fig.2: Transcriptome assembly for poly(A)-enriched from a human cerebral cortex developmental time course.** (**A**) Schematic diagram of the *SOX1-OT* locus in ReN cells with indicated exons (numbered boxes) and TSS (bent arrows). (**B**) IGV visualization of the transcriptome assembly for human developing cortex samples at gestation week 13 (T1), 16 (T2), 21 (T3) and 23 (T4), compared to the *SOX1-OT* variants detected in ReN cells.

The Sox1 region is indicated by green vertical lines; red exclamation marks indicate transcript isoforms that are antisense relative to the annotated *SOX1*; red arrows indicate the *SOX1-OT* isoforms detected in the RNA-seq dataset which contain a similar exon structure as the ones detected by our RT-PCR and 3’RACE; red boxes highlight regions within *SOX1-OT* variants which differ markedly between the isoforms detected by RNA-seq and RT-PCR; green arrows depict *SOX1-OT* isoforms detected by 5’RACE and by RNA-seq in the developing cortex.

**Supplementary Fig.3: Expression profile of *SOX1-OT* in cancer cell lines.** (**A**) Schematic diagram of the *SOX-1OT* locus in ReN cells with indicated exons (numbered boxes) and TSS (bold bent arrows). (**B**) Snapshot images of the *SOX1* locus on human chromosome 13 taken from the UCSC genome browser (hg19) showing the Refseq and UCSC gene annotations (blue), followed by the tracks of the CpG islands (green), and the RNAseq data from ENCODE available data for Hela (light blue) and MCF7 (black) cells. These annotations suggest that, consistent with our RT-PCR data (Figure 7), Hela cells are negative in this region whilst some transcription is detected in MCF7 cells.

**Supplementary Fig.4: Variety of Poly(A)-signals at the SOX1-OT** **locus in the brain.** (A) Schematic diagram of the SOX1-OT locus in ReN cells with indicated exons (numbered boxes) and TSS (bold bent arrows). Snapshot images of the SOX1 overlapping transcript loci on human (hg19 chr13:112626600-112766000) (B) and mouse (mm9, chr8: 12,300,135-12,439,035) (C) taken from the UCSC genome browser showing the currently annotated structures of these transcripts in the two species and Poly(A)-seq from [7]. The turquoise region highlights detected Poly(A)-signals in the 3’UTR of SOX1, while the yellow and red regions highlight Poly(A) sites detected in human brain samples.

**SUPPLEMENTARY METHODS:**

**Mouse stem cell samples**

Mouse embryonic stem cells (mESC) and mouse neural stem cells (mNSC) were cultured as described elsewhere [1] and [2], respectively) and used to prepare RNA as previously described.

**RNA sequencing data analysis**

Publicly available datasets were downloaded from the European nucleotide archive (ENA, [www.ebi.ac.uk/ena](http://www.ebi.ac.uk/ena)). The dataset consists of stranded paired-end RNA-seq dataset from the developing human cortex [3] bulk tissue [4] (Array Express: E-GEOD-71315) for 4 developmental timepoints of human cortex development: gestation week (GW) 13, 16, 21 and 23, in duplicates. After read mapping and transcriptome assembly using the human reference genome, the transcriptomes for each developmental timepoint were merged with StringTie [5]. For the earliest timepoint, since no biological replicates were available the transcriptome assemblies for GW13 and GW14.5 were combined. For each developmental timepoint transcriptome assemblies were generated for RNA-seq data obtained from Poly(A)-enriched RNA samples and rRNA-depleted total RNA samples. The obtained transcriptomes were merged for the biological replicates and visualised using the IGV browser [6].

**Poly(A)-signal analysis**

Processed bed files for PolyA-seq produced by [7] were downloaded from ArrayExpress (E-GEOD-30198) and uploaded as custom tracks to the UCSC genome browser to visualise the detected Poly(A)-signals in human and mouse. The data was processed by the original authors to show high confidence Poly(A)-signals (_align_ tracks) and peaks of Poly(A)-signals within 30bp on the same strand (_cluster_ tracks). The latter was performed as poly(A) sites generally correspond to one major peak surrounded by minor peaks reflecting likely the inexact nature of transcript termination. Kidney samples were used as negative controls.

**Supplementary table 1:** Sequences for forward (F) or reverse (R) primers used in this study.

| Primer | Sequence (5`-3`) |  |  |  |
| --- | --- | --- | --- | --- |
| F1 | TGGAAGTTTCACTCAGCCGT |  |  |  |
| F2 | CTTGGCATCTTCTTCCGAGCA | | | |
| F2A | TGGGCAGGCAGGACTTCA |  |  |  |
| F4 | ACCAGAGCCGAGGACTAAAC | | |  |
| F4a | GACCAGAGCCGAGGACTAAAC |  |  |  |
| F5a | ACCACTCCATTGCAGAAAAGC | | | |
| F6 | CCACCCGGTCCGGAATGA |  |  |  |
| F6a | GCAGAGCGTTAGGGGCG | | |  |
| F7 | TCACTTATCTGGAAACCTGCGG |  |  |  |
| F7a | ATCTGGAAACCTGCGGTTGG | | |  |
| F11 | ATGTGCAGGACTAAGGCGAC |  |  |  |
| F11a | CTGCGACCACCTACCATCAC | | |  |
| F12 | ACCCAGGAAAAAGCTACGGG |  |  |  |
| F13 | TACAGCCCCATCTCCAACTC | | |  |
| R1 | GATAATGACCCCCGGTTCCC |  |  |  |
| R2 | GCATGGGCACGACTTGG | | |  |
| R3 | TTGTTGGTTGCACTACCCCT |  |  |  |
| R4 | GCACTACCCCTTCACATCCT | | |  |
| R4a | TTCACATCCTACCCCCTCCTT |  |  |  |
| R5 | TCAATGTTTATTTCGACTTCCCG | | | |
| R5a | TTATTTCGACTTCCCGGGGC |  |  |  |
| R6 | TTACAGTTAGTTCCTCCTCCAGC | | | |
| R6a | ACAGTTAGTTCCTCCTCCAGCTC |  |  |  |
| R7 | CTGCGGATTGCAGCGAC | | |  |
| R7a | CGTTCGCTGCGGATTGC |  |  |  |
| R11 | GTAGGTGGTCGCAGTGAGAG | | |  |
| R11a | CTTGCAACTTCCGTGACCAA |  |  |  |
| R12 | GACCTCTGCATCCCCTCAAC | | |  |
| R13 | GCTCCGACTTCACCAGAGAG |  |  |  |

**REFERENCES:**

1. Tufarelli C, Stanley JA, Garrick D, Sharpe JA, Ayyub H, Wood WG, Higgs DR. (2003) Transcription of antisense RNA leading to gene silencing and methylation as a novel cause of human genetic disease. Nat Genet. 34:157-65.
2. Alcock J, Sottile V (2009). Dynamic distribution and stem cell characteristics of Sox1-expressing cells in the cerebellar cortex. Cell Res. 19:1324-33.
3. Liu SJ, Nowakowski TJ, Pollen AA, Lui JH, Horlbeck MA, Attenello FJ, et al. (2016) Single-cell analysis of long non-coding RNAs in the developing human neocortex. Genome Biol. 17:67.
4. Sauvageau M, Goff LA, Lodato S, Bonev B, Groff AF, Gerhardinger C, et al. (2013) Multiple knockout mouse models reveal lincRNAs are required for life and brain development. Elife 2:e01749.
5. Pertea M, Pertea GM, Antonescu CM, Chang TC, Mendell JT, Salzberg SL, et al. (2015) StringTie enables improved reconstruction of a transcriptome from RNA-seq reads. Nat. Biotechnol. 33:290–5.
6. Thorvaldsdóttir H, Robinson JT, Mesirov, JP. (2013) Integrative Genomics Viewer (IGV): high-performance genomics data visualization and exploration. Brief Bioinform. 14:178–92.
7. Derti A, Garrett-Engele P, Macisaac KD, Stevens RC, Sriram S, Chen R, et al. (2013) A quantitative atlas of polyadenylation in five mammals. Genome Res. 22:1173-83.
