# Supplementary figures and images for: Expression of a SOX1 overlapping transcript in neural differentiation and cancer models
Source: Cell Mol Life Sci. 2017 Jul 3;74(22):4245–58. doi: 10.1007/s00018-017-2580-3 (PMC5641280; doi:10.1007/s00018-017-2580-3)

A

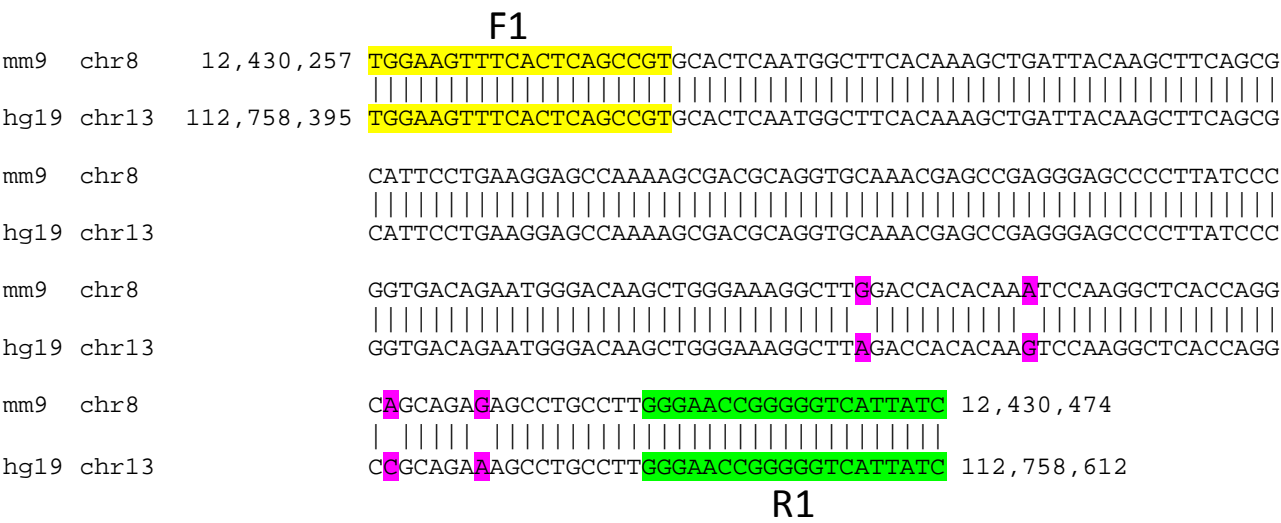

B

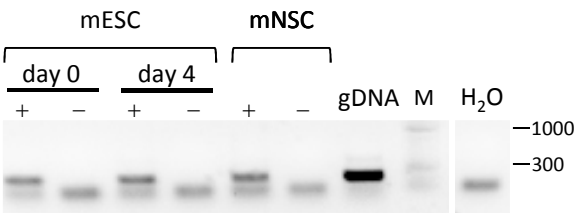

C

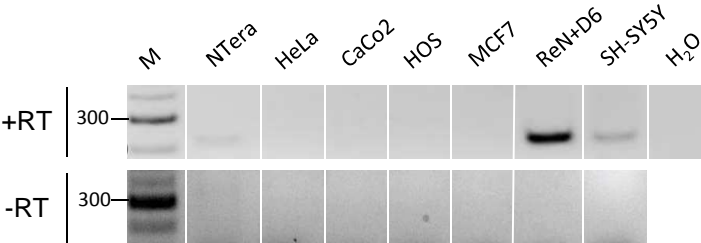

Supplement: Supplementary file 2 — Supplementary material 2 (PDF 75 kb) [file 18_2017_2580_MOESM2_ESM.pdf]

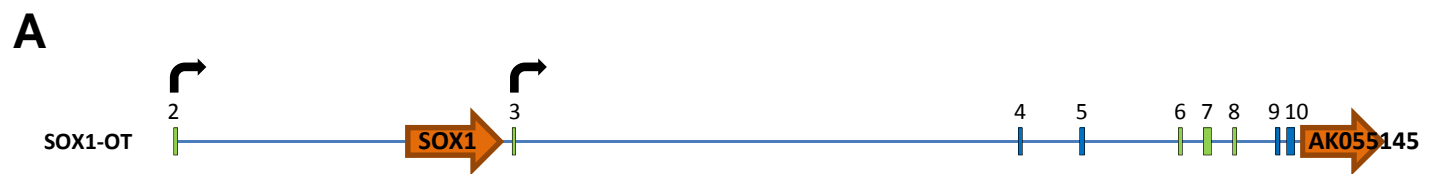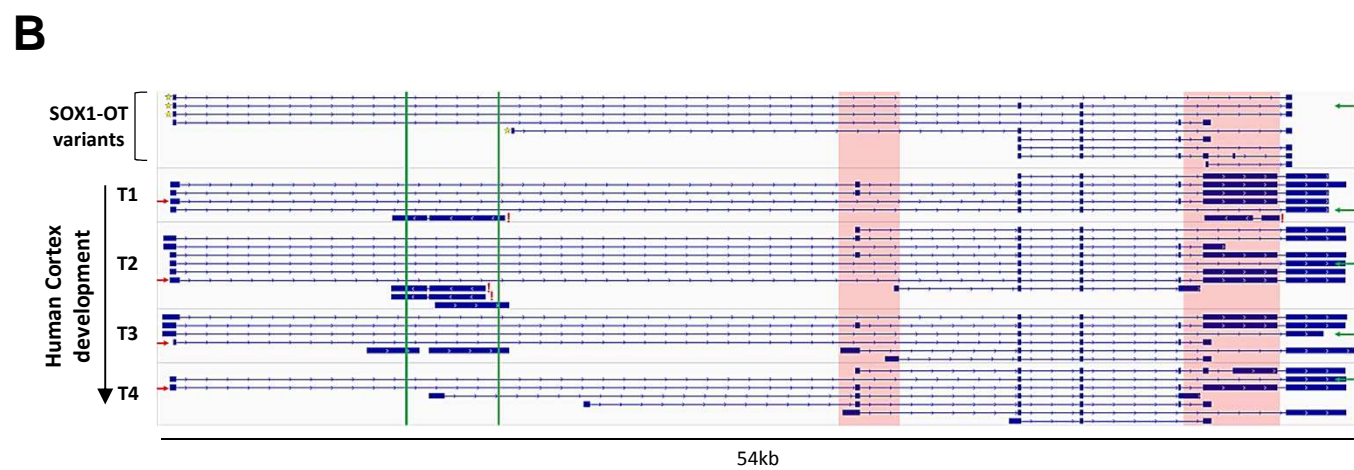

Supplement: Supplementary file 3 — Supplementary material 3 (PDF 163 kb) [file 18_2017_2580_MOESM3_ESM.pdf]

**A**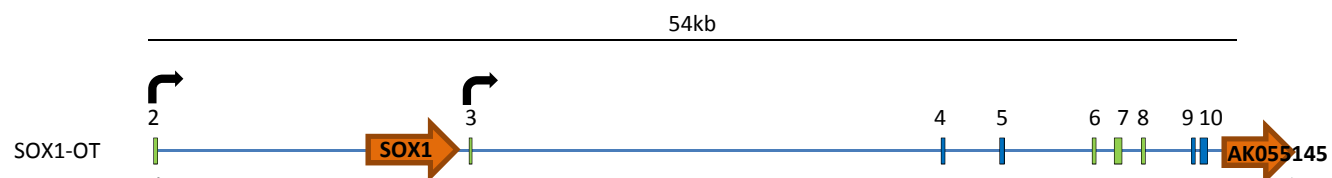**B**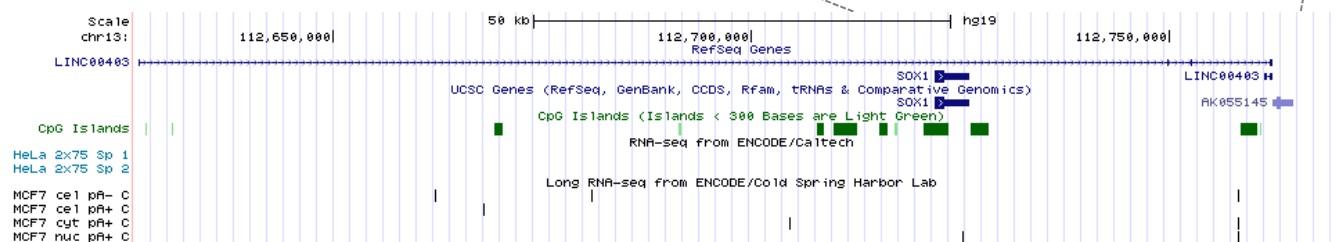

Supplement: Supplementary file 4 — Supplementary material 4 (PDF 71 kb) [file 18_2017_2580_MOESM4_ESM.pdf]

Supplementary Figure 4

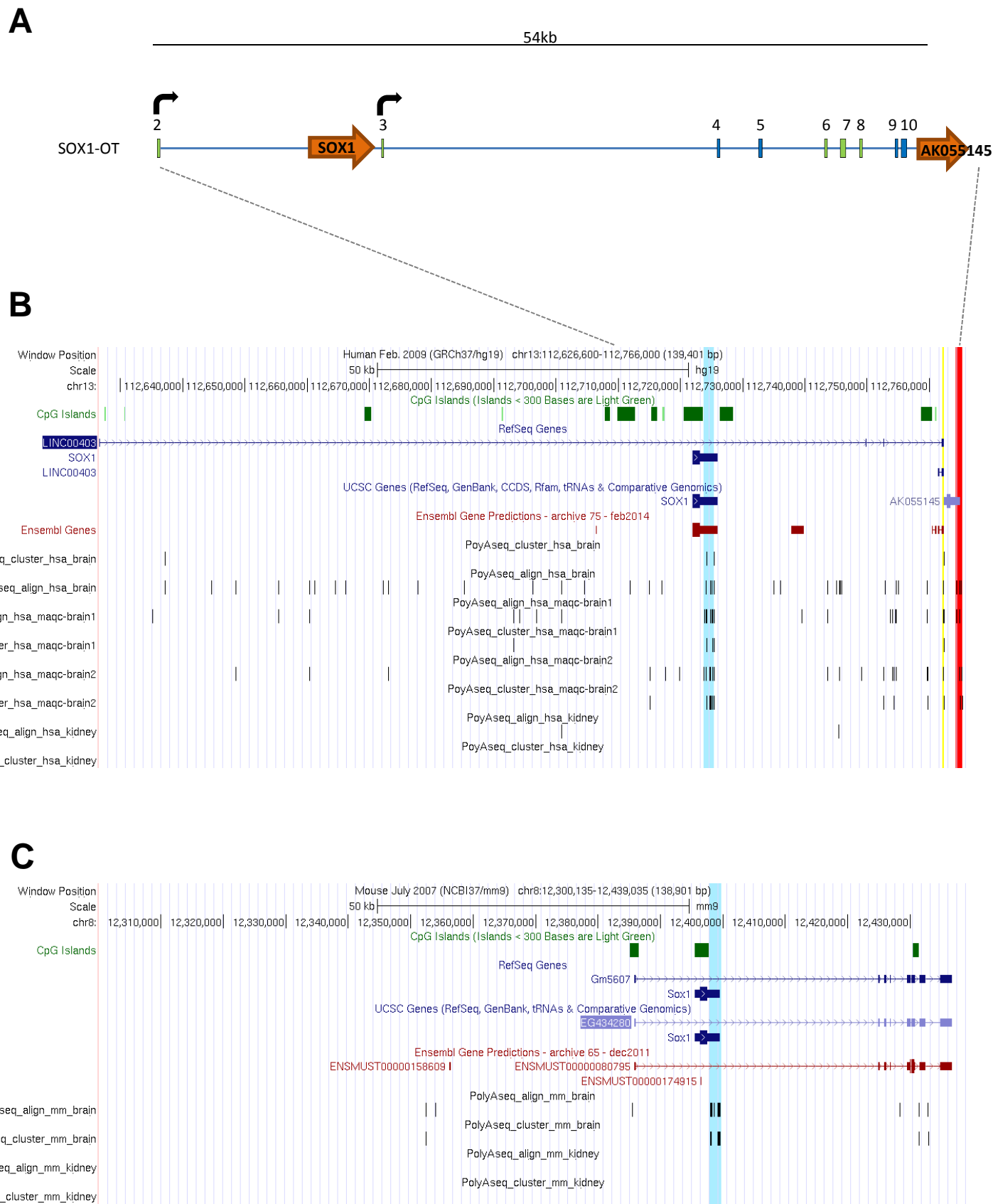

Supplement: Supplementary file 5 — Supplementary material 5 (PDF 96 kb) [file 18_2017_2580_MOESM5_ESM.pdf]
